# Evidence of a novel sublineage of Streptococcus agalactiae in elephants from zoo populations in Germany

**DOI:** 10.1099/mgen.0.001489

**Published:** 2025-09-04

**Authors:** Linda Fenske, Elita Jauneikaite, Maria Getino, Yu Wan, Alexander Goesmann, Tobias Eisenberg

**Affiliations:** 1 Bioinformatics and Systems Biology, Justus Liebig University Giessen, Giessen, Germany; 2 NIHR Health Protection Research Unit in Healthcare Associated Infections and Antimicrobial Resistance, Department of Infectious Disease, Imperial College London, London, UK; 3 Department of Infectious Disease Epidemiology, School of Public Health, Imperial College London, London, UK; 4 David Price Evans Global Health and Infectious Diseases Research Group, University of Liverpool, Liverpool, UK; 5 Department of Veterinary Medicine, Hessian State Laboratory, Giessen, Germany

**Keywords:** capsule typing, elephants, MLST, penicillin-binding proteins, prophage regions, *Streptococcus agalactiae*, virulence, whole-genome sequencing

## Abstract

*Streptococcus agalactiae* research primarily centres on investigating human and bovine infections, although this pathogen also can be carried and cause infections in a wider range of animal species. Moreover, infections with *S. agalactiae* are posing significant health implications, and recent studies furthermore are highlighting a potential zoonotic risk. Despite the relatively frequent isolation of *S. agalactiae* from elephants, only a few reports document infections in wild and zoo populations. We performed a comparative genomic analysis of 24 elephant isolates from three different zoos in Germany to achieve a comprehensive characterization. Elephant isolates showed pronounced phylogenetic divergence from isolates of other host species, while also forming clusters based on zoo of origin and their genotypes (MLST profiles). Capsular serotypes could not be predicted for the majority of the isolates (*n*=20/24). Several genes, exclusively associated with the elephant host, may underlie the pathogen’s capacity to improve its survival and virulence across varied ecological niches. This study not only deepens our understanding of *S. agalactiae* across diverse species and environments but also represents the first whole-genome sequencing characterization of *S. agalactiae* isolates from elephants, helping to expand our knowledge about infections in animals.

**Impact Statement**

This study provides the first whole-genome characterization of *Streptococcus agalactia*e isolates from elephants, revealing their distinct phylogenetic divergence from group B streptococcus (GBS) isolates of other host species. Our findings suggest that elephant-derived GBS may represent a unique sublineage, with potential adaptations to their host and environment. The absence of identifiable capsular serotypes in most isolates and the presence of host-associated genes highlight the need for further research on the pathogen’s evolution, virulence and zoonotic potential. Expanding genomic studies to include isolates from broader geographic regions will be crucial for understanding the role of GBS in exotic animals and its potential impact on both wildlife and public health.

## Data Summary

Raw whole-genome sequencing data for elephant isolates used in this study are available in the European Nucleotide Archive (ENA; https://www.ebi.ac.uk/ena/browser/home) project accession number PRJEB28328, and individual GBS isolate accession numbers have been listed in Table S1, available in the online Supplementary Material. Reads and assemblies from Oxford Nanopore MinION sequencing for two GBS isolates, 161002207-5 and 161002207-6 have been deposited to ENA project accession number PRJNA1244849.

## Introduction

*Streptococcus agalactiae*, commonly known as group B streptococcus (GBS), is a facultative anaerobic, non-motile, chain-forming, Gram-positive, catalase-negative bacterium that can cause infections in a wide range of hosts. As a human pathogen, GBS plays a significant role as the primary cause of neonatal infections, usually transmitted from mother to newborn during childbirth, provoking conditions such as pneumonia, meningitis or septicaemia [1]. Though GBS is also known to be carried asymptomatically within the urogenital tract and the gut, it can also cause infections in adults, especially the elderly. Examples of such infections are urinary tract infections, diabetic foot infections, osteomyelitis and, in rare cases, even more severe conditions like toxic shock syndrome, necrotizing fasciitis, disseminated intravascular coagulopathy and renal dysfunction [2,3]. GBS is also well known as a pathogen in dairy farming, where it was first reported as a causative agent for the majority of mastitis cases in 1927 [4]. The implementation of the five-point hygiene plan in the 1960s, which included measures such as rapid identification and treatment of infected cows, whole-herd antibiotic dry cow therapy, post-milking teat disinfection, culling chronically infected cows and routine disinfection of milking equipment, significantly reduced the prevalence of GBS infections but has not eliminated its relevance in the dairy industry today. Although GBS research predominantly focuses on human and bovine diseases, GBS has also been reported to cause infections in a variety of animal species including dolphins [5], camels [6], rats [7], fish, seals and other aquatic species [8,9] and elephants [10].

One of the key virulence factors in GBS is the capsular polysaccharide, which is involved in host immune response evasion [11]. Based on the antigenic properties of the polysaccharide capsule, a serotyping system for GBS was developed [12]. Currently, ten major serotypes (Ia, Ib and II-IX) are recognized, encoded by the capsular locus (*cps*), which comprises 16–18 genes [13,14]. It is known that the prevalence and distribution of serotypes vary across geographic regions, host species and clinical presentations [15], and some of the serotypes are also associated with different virulence potential [16,17]. Most studies on GBS focus on human isolates, and though studies in animals have been published, there is a lack of detailed genomic description of GBS isolated from elephants. The earliest report of GBS in elephants dates back to 1997, when the bacterium was identified in pododermatitis lesions of African elephants [18], followed by its detection in Asian elephants in 2008 [19]. Since then, only one other study reported GBS isolated from African and Asian elephants in zoos in Germany [10]. The latter study employed molecular techniques to characterize and compare the identified GBS isolates from elephants to those in other animals and humans, highlighting the potential genetic diversity of GBS causing infections in elephants, including the main finding that most of the GBS from elephants were non-typable using the standard sera or capsular locus typing by multiplex PCR. The non-typable state of GBS isolates is of interest as disease-causing GBS are typically encapsulated, though some studies have reported isolates from humans lacking a capsular locus [20], and GBS isolates from animals have been reported as non-typable [21] or not clearly typable due to previously unknown serotype variants. This could be due to the multiplex PCR designed for human-specific GBS serotypes that does not account for the potential capsular loci diversity of GBS from animals [22].

In the present study, we conducted a comparative genomic analysis of 24 GBS isolates obtained from African and Asian elephants between 2010 and 2023 from zoos in Germany. We aimed to characterize in detail the genomes of these GBS isolates by determining their virulence factors, genotypes based on multilocus sequence typing (MLST), capsular serotypes and potential host-specific genes. This provides valuable insights into the evolution and pathogenicity of GBS populations in less studied host species such as elephants. To our knowledge, this is the first study characterizing GBS isolates from elephants using whole-genome sequencing (WGS).

## Methods

### Bacterial isolates

A total of 24 GBS isolates from African and Asian elephants in German zoos were whole-genome sequenced and analyzed. Of these, 23 isolates were obtained during routine bacteriological investigations from elephants in two different zoos (A+B; ~200 km apart) between 2010 and 2016. Twelve of these isolates were obtained from the previous study by Eisenberg *et al*. [10], indicated in Table S1. One isolate (IHIT53690) was obtained from an elephant in Zoo C (~400 km apart from Zoo A; ~200 km apart from Zoo B) in 2023 as part of a routine investigation carried out by the Institute of Hygiene and Infectious Diseases of Animals, Giessen. Metadata of all draft genomes used in this study are provided in Table S1.

### Identification by MALDI-TOF MS

Bacterial isolates were selected from the culture plates and then transferred to steel targets according to the manufacturer’s instructions (Bruker Biotyper, Bruker Daltonics, Bremen, Germany). Isolates were prepared using the direct smear method and analysed by MALDI-TOF MS using Biotyper (v3.3.1.0).

### WGS, raw reads processing and assembly

GBS isolates were streaked on Columbia blood agar plates (Oxoid, Basingstoke, UK) and incubated at 37 °C, 5% CO_2_ overnight. Genomic DNA was extracted using the GenElute bacterial Genomic DNA kit (Sigma-Aldrich, Burlington, MA, USA) following the manufacturer’s instructions for Gram-positive bacteria with modifications as follows: GBS was lyzed in 180 µl G+lysis solution with added 20 µl mutanolysin (Sigma-Aldrich, USA; prepared at 3,000 U ml^−1^) and 20 µl lysozyme (Sigma-Aldrich, USA; prepared at 100 mg ml^−1^) added prior to incubation at 37 °C for 1 h. Subsequent steps followed the manufacturer’s instructions. DNA was quantified using a NanoDrop spectrophotometer (Thermo, Waltham, MA, USA).

For short-read sequencing, multiplexed DNA library preparation was conducted according to the Illumina protocol, and WGS was performed on a HiSeq X Ten system (Illumina, USA) with 150-cycle paired-end mode. Raw reads were trimmed and filtered with fastp (v0.23.2) [23], and reads were checked for quality with FastQC (v0.11.9) (www.bioinformatics.babraham.ac.uk/projects/fastqc/). Trimmed reads were used for *de novo* assembly into contiguous sequences using Unicycler (v0.5.0) [24]. The draft assemblies were purged from possible errors with Polypolish (v0.5.0) [25] and POLCA from the MaSuRCA (v4.1.0) (Maryland Super Read Cabog Assembler) genome assembly and analysis toolkit [25].

Two isolates (161002207-5, 161002207-6) were selected for long-read sequencing using an Oxford Nanopore Technologies (ONT, UK) MinION Mk1B device. Genomic DNA libraries were prepared using the Rapid Barcoding Sequencing Kit (SQK-RBK004; ONT, UK) and loaded into an R9.4.1 flow cell (FLO-MIN106; ONT, UK). Basecalling was conducted using Guppy (v6.5.7) (ONT, UK) under its super-accuracy mode. Long reads were checked for quality using Filtlong (v0.2.1) (https://github.com/rrwick/Filtlong) and assembled using Trycycler (v0.5.5) [26] followed by short-read polishing using Polypolish and pypolca (v0.3.1) [27,28] as recommended in Ryan Wick’s guide to bacterial genome assembly [29]. Polished assemblies were annotated using Bakta (v1.7.0) [30]. Contamination and completeness of all genome assemblies were estimated with CheckM2 (v1.0.1) [31], and furthermore, a taxonomic verification with GTDB-Tk (v2.2.3) was conducted [32].

### Population analysis

MLSTs were assigned using mlst (v2.23.0) (https://github.com/tseemann/mlst) utilizing the PubMLST *S. agalactiae* database [33] (https://pubmlst.org/organisms/streptococcus-agalactiae). Comparative genome analyses for determination of the pan and core genome, as well as singleton genes, were performed with EDGAR (v3.2) [34]. For the creation of the phylogenetic tree, the core genes of all genomes were computed. In the following step, alignments of each core gene set are generated using MUSCLE, and the alignments are concatenated to one huge alignment. The tree was constructed with FastTree using the approximately maximum likelihood method. For visualization of the core genome phylogeny, iTOL (v7) was used [35]. For phylogenetic placement of the elephant-derived isolates, a subset of GBS genomes from different host species was selected and included for comparison. Up to 23 representative genomes for each of the different host species were selected from a database of confirmed GBS genomes [36]. These included genomes from rats (*n*=5), dogs (*n*=4), dolphins (*n*=1), fish (*n*=23), frogs (*n*=2), seals (*n*=4), camels (*n*=16), bovines (*n*=17) and humans (*n*=23) (Table S1). To validate the findings of the phylogenetic analysis, a target-free split *k*-mer analysis and single-linkage clustering were conducted using SKA (v1.0) [37]. Specifically, *k*-mer files (*k*=15) were generated with the fasta subcommand under default parameters; pairwise SNP distances were calculated, and SKA clusters were defined at a threshold of 10 SNPs, if they met the minimum identity cutoff of 0.9.

### Antimicrobial resistance genes and mobile genetic elements

Antimicrobial resistance (AMR) genes were detected using AMRFinderPlus [38], the Resistance Gene Identifier software [39] and abriTAMR (https://github.com/MDU-PHL/abritamr). Putative resistance markers to *β*-lactam antibiotics were analysed using EDGAR. Therefore, orthologous genes encoding the penicillin-binding proteins PBP1A, PBP1B, PBP2A, PBP2B and PBP2X were identified in the draft genomes. The amino acid sequences of these penicillin-binding protein (PBP) genes were visualized and manually arranged in Jalview [40] based on sequence similarities and subsequently compared to the penicillin-susceptible strain 2603 V/R (GenBank: NC_004116). For reconstruction and typing of potential plasmids, MOB-suite was used (v3.1.8) [41], and the PHASTEST web tool was used to identify prophage regions within the draft genomes [42]. For 12 isolates, capsular serotyping and antimicrobial susceptibility testing results from Eisenberg *et al*. [10] were incorporated in Table S2.

### Analysis of the capsular locus genes

A combination of GBS-SBG [43], srst2 (v0.2.0) [44], seq_typing (v2.3.0) (https://github.com/B-UMMI/seq_typing) and KMA (v1.4.12) [45] was used to try to determine the capsular serotype based on the capsular loci genes present. As the majority of isolates remained untypable despite utilizing various tools, an in-depth analysis of the *cps* locus was conducted. The *cps* loci of the elephant-derived GBS isolates were extracted with *in silico* PCR utilizing SnapGene (v8.0.2) (https://www.snapgene.com/). The primers used were introduced in a previous study [22]. If no binding sites for these primers were detected within the genomes, alternative primers flanking the *cps* locus at more distant regions were used [20].

### Genome-wide association studies

A gene-enrichment analysis, focused on GBS genomes from elephants, was performed using Scoary (v1.6.16) [46] from the gene presence/absence matrix generated by Panaroo (v1.5.0) [47]. The latter was generated from the GFF3 files annotated with Bakta. The host groups were defined as binary phenotypes with a value of 1 assigned to genomes from the elephant host group and 0 to those GBS genomes from all other species.

## Results

### Sequencing statistics

**Fig. 1.**
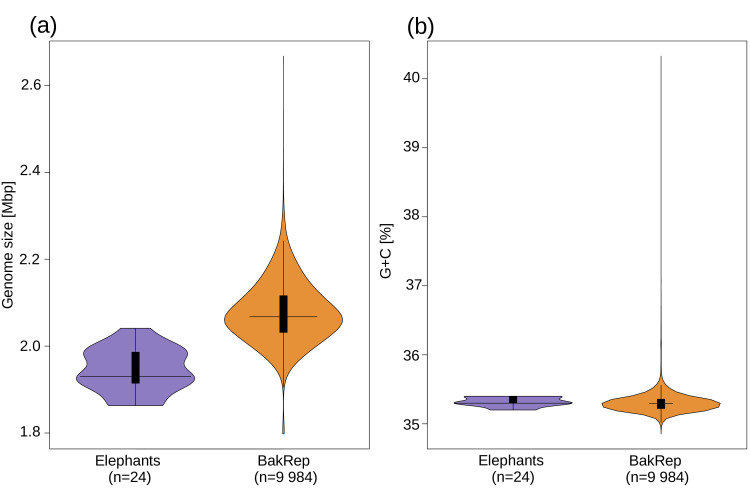
Comparison between genome size (**a**) and G+C content (**b**). GBS isolates from elephants used in this study (*n*=24, purple) and all GBS genomes included in BakRep (*n*=9,984, orange). Horizontal black lines in the middle indicate medians; bold black bars represent interquartile ranges; vertical black lines represent outliers.

All 24 genomes were classified as *Streptococcus agalactiae* according to the GTDB taxonomy. The estimated completeness of all draft genomes was above 99.99% with a contamination rate lower than 0.07%. The combined lengths of the assembled contigs range from 1,863,022 to 2,041,196 bp with a G+C content between 35.4 and 35.5 mol%. To evaluate how the genome size and G+C content of the elephant isolates used here integrate into the overall context of GBS genomes, we compared the elephant draft genomes to all GBS genomes currently included in the bacterial web repository BakRep [48]. The BakRep v1 contains 10,359 genomes classified as *S. agalactiae* (as of October 2024). After filtering available genomes for completeness of >95% and contamination rate <1%, 9,984 genomes remained for comparison. As none of the genomes included in the repository listed the elephant as the host species, no further subdivision of the data was made. The GBS genomes included in BakRep had a genome size ranging from 1,798,114 to 2,667,783 bp (only two genomes were larger than 2.5 Mbp, i.e. 2.53 and 2.67 Mbp, respectively, in the whole collection) with a G+C content ranging from 34.9 to 40.3 mol% (Fig. 1). This highlights that the GBS draft genomes from elephants have a mean genome size of 1,930,136 (sd±515,960.30) that is below the genomes contained in BakRep with a mean genome size of 2,067,641 (sd±6,982.26).

### Characteristics of GBS genomes from elephants: genotypes, capsular types and AMR

Out of 24 GBS isolates from elephants, 13 (54.17%) were assigned to ST2019, 10 isolates were assigned to sequence type ST2304 and 1 isolate was assigned to ST2305. The latter two STs were first reported in this study and submitted to the *S. agalactiae* MLST database (Table 1). Interestingly, all ST2019 GBS isolates (*n*=13) came from Zoo A, all ST2304 GBS (*n*=11) came from Zoo B and the one GBS ST2305 isolate came from Zoo C; of note, ST2305 was a single-locus variant (SLV) of ST2304. None of the identified STs could be assigned to a clonal complex.

**Table 1.** MLST results for 24 isolates of *S. agalactiae* from elephants analyzed in this study utilizing the MLST scheme of Jones *et al*. [74]. The newly identified STs in this study are highlighted in bold

Isolate	Allele							ST
	*adhP*	*pheS*	*atr*	*glnA*	*sdhA*	*glcK*	*tkt*	
10-7-D-02041; 141000096/2; 141014875; 151001913-2; 151002450-1; 151002450-2; 151003337-1; 151003337-2; 151003441-1; 151003441-2; 151003441-3; 151008126; 151010216	20	13	15	1	15	9	5	2019
161002207-5; 161002207-6; 161002208-5; 151004802; 151005628-1; 151005628-2; 151006965-1; 151006965-2; 151006967; 151010127	16	17	4	2	4	9	5	**2304**
IHIT53690	16	23	4	2	4	9	5	**2305**

We knew from a previous study [10] that 12 of our elephant-derived GBS isolates were reported as non-typable for their capsular serotype using molecular capsular typing methods. The remaining 12 isolates were not routinely serotyped in the laboratory. After WGS, 4 out of 24 (16.7%) GBS isolates were assigned to serotype Ia. For all other isolates, no clear assignment could be made even after using various accepted GBS capsular typing methods (Table 2).

**Table 2.** Serotyping results for 24 isolates of *S. agalactiae* from elephants obtained with different *cps* typing tools

Isolate	Tool		
	GBS-SBG (best match)	Seq_typing (best match)	KMA (best match)
161002207-5	nt	nt	VI (score: 15264; coverage: 1.30%)
161002207-6	Ia (coverage: 99.98%)	Ia (sequence covered: 95.74%)	Ia (score: 1111688; coverage: 85.85%)
161002208-5	III-3 (coverage: 78.85%)	Ia (sequence covered: 97.03%)	Ia (score: 1305804; coverage: 87.78%)
10-7-D-02041	nt	nt	VI (score: 12116; coverage: 1.28%)
141000096/2	nt	nt	VI (score: 13035; coverage: 1.27%)
141014875	nt	nt	VI (score: 13505; coverage: 1.28%)
151001913-2	nt	nt	II (score: 12165; coverage: 2.35%)
151002450-1	nt	nt	VI (score: 15747; coverage: 1.29%)
151002450-2	nt	nt	VI (score: 19190; coverage: 1.28%)
151003337-1	nt	nt	VI (score: 14669; coverage: 3.12%)
151003337-2	nt	nt	VI (score: 12245; coverage: 1.26%)
151003441-1	nt	nt	VI (score: 16389; coverage: 1.29%)
151003441-2	nt	nt	VI (score: 11571; coverage: 1.29%)
151003441-3	nt	nt	VI (score: 16338; coverage: 1.34%)
151004802	nt	nt	VI (score: 14957; coverage: 1.29%)
151005628-1	nt	nt	VI (score: 17042; coverage: 1.29%)
151005628-2	nt	nt	VI (score: 15494; coverage: 1.34%)
151006965-1	nt	nt	VI (score: 13236; coverage: 1.29%)
151006965-2	Ia (coverage: 99.99%)	Ia (sequence covered: 97.03%)	Ia (score: 1127495; coverage: 85.73%)
151006967	nt	nt	VI (score: 12073; coverage: 1.27%)
151008126	nt	nt	VI (score: 18202; coverage: 1.29%)
151010127	Ia (coverage: 100%)	Ia (sequence covered: 96.65%)	Ia (score: 1139070; coverage: 86.66%)
151010216	nt	nt	VI (score: 15068; coverage: 1.27%)
IHIT53690	VII (coverage: 56.55%)	IX (sequence covered: 65.85%)	IV (score: 995859; coverage: 80.59%)

**Fig. 2.**
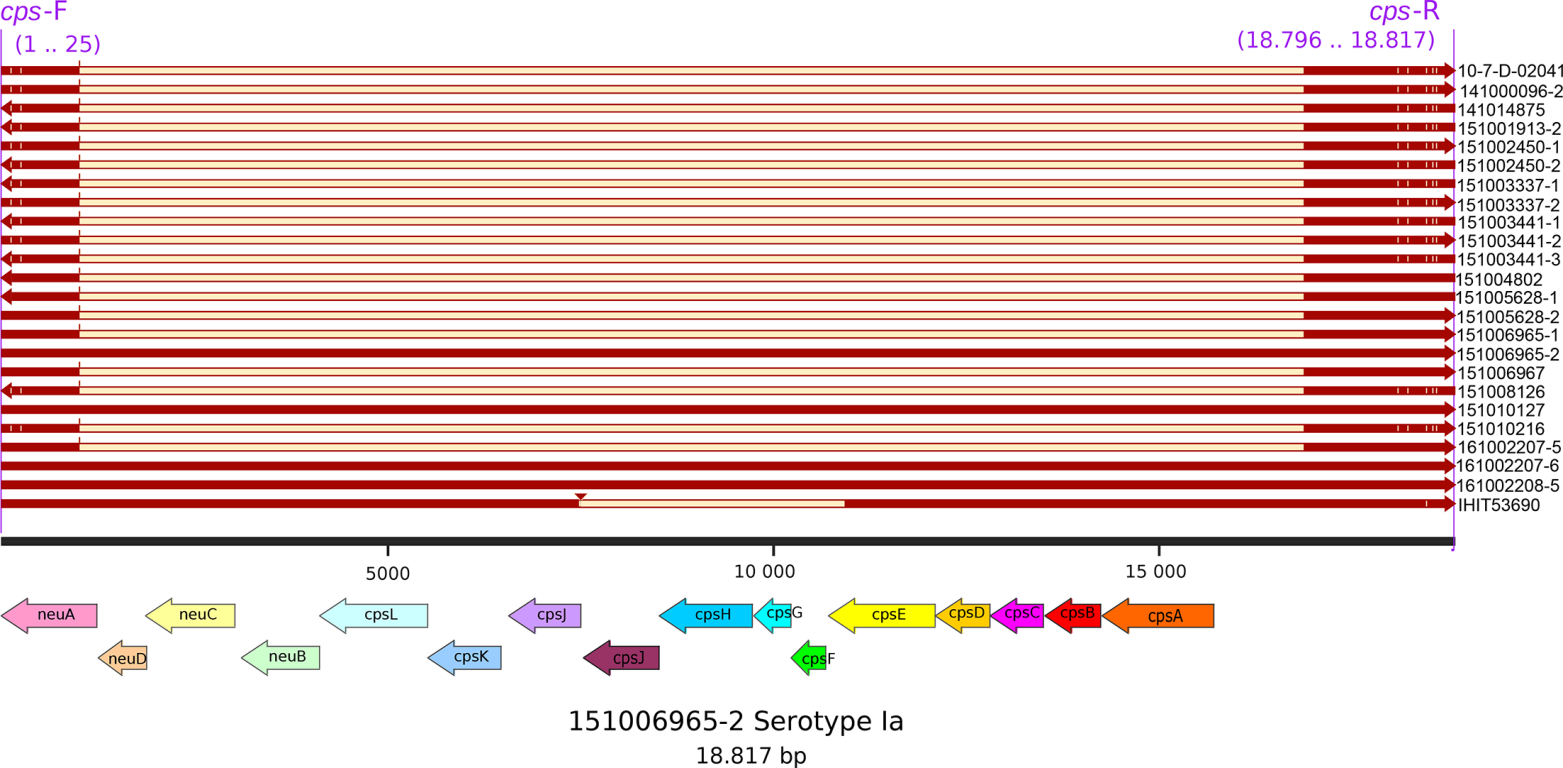
Multiple-sequence alignment of the extracted *cps* locus of the elephant-derived GBS isolates. The regions between the primers (*cps-*F and *cps*-R) are indicated in dark red, while the deletion is shown in beige. Coloured arrows at the bottom depict the genetic organization of the serotype Ia gene sequence from isolate 151006965-1, which was used as reference. Primer binding sites are indicated at the top in purple. The figure was created with SnapGene (v8.0.2), and annotation of the genes was performed with Bakta (v1.7.0).

To analyse the *cps* locus in greater detail, *cps* loci of all elephant-derived isolates were extracted. Binding sites for the primer sequences used by Crestani *et al*. [22] could only be found in the isolates 151006965-2, 151010127, 161002207-6, 161002208-5 and IHIT53690. All these isolates, except IHIT53690, matched the serotype Ia reference sequence (GenBank: LT671983.1) with high similarity, aligning with the results from other applied methods. The region extracted using primer sequences from Creti *et al*. [20] exhibited a deletion in the remaining 19 isolates, similar to the one previously reported by Creti *et al*. (Fig. 2). For IHIT53690, no clear serotype assignment could be made due to several gaps and mismatches (Fig. S2).

Analysis of AMR determinants showed that all 24 GBS isolates likely remain susceptible to most antibiotics due to no acquired resistance genes detected in the genomes, bar *mprF* gene. In PBP1A, all isolates shared a V742A substitution and a deletion of four amino acid residues (739–742) previously described but not associated with reduced susceptibility to *β*-lactams [49]. In PBP1B, all isolates had an A95D substitution, already detected in GBS from Japan associated with reduced penicillin resistance [50], and in 13 out of 24 (54.17%) isolates, a V64I substitution was identified (not previously described). In the PBP2A, 13 out of 24 (54.17%) isolates had the E18K substitution, and 10 out of 24 (41.76%) had the S394G substitution (not previously described). All isolates showed the V80A substitution in the PBP2B, which was previously detected in other studies [49,51], while 13 out of 14 (54.17%) isolates had a D572N substitution in the PBP2X gene (not previously described). Notably, the 13 isolates with the E18K substitution in PBP2A and the D572 substitution in PBP2X all originated from Zoo B, while the 10 isolates with the S394G substitution came from Zoo A. However, phenotypic testing done in the previous study by Eisenberg *et al*. [10] confirmed high susceptibility to penicillin G and other *β*-lactams, including those with identified PBP mutations. Additionally, 16 of the 24 tested isolates exhibited low-level phenotypic resistance to gentamicin (Table S2). All substitutions and results of previously done antimicrobial susceptibility testing are listed in Table S2.

### Mobile genetic elements: prophages identified in GBS isolates

**Fig. 3.**
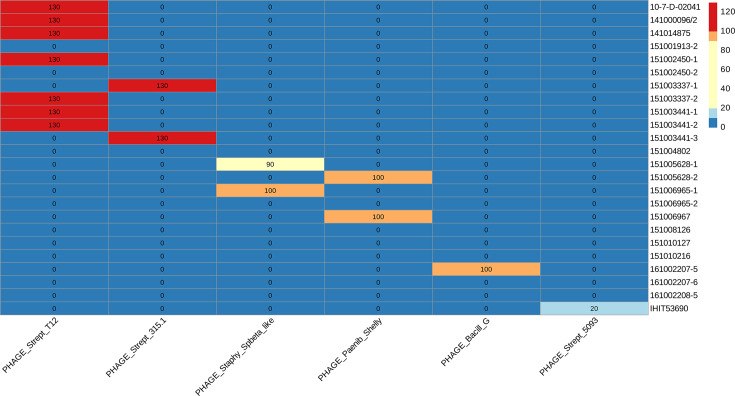
Heatmap of the identified prophage regions. The score (<70: incomplete; 70–90: questionable; >90: intact) for each isolate is indicated in the boxes. The heatmap was created with R from the results generated with PHASTEST.

There were neither complete nor partial plasmid sequences identified in any of the 24 isolates. The prophage analysis suggested that 13 out of 24 (54.17%) isolates had predicted intact phages: 7 out of 24 (29.17%) isolates had *Streptococcus* phage T12 (NC_028700), 2 out of 24 (8.33%) isolates had *Streptococcus* prophage 315.1 (NC_004584.1) and 1 out of 24 (4.17%) had an intact *Staphylococcus* phage SPbeta-like (NC_029119.1), while another isolate carried a questionable version of the same phage. Two out of twenty-four (8.33%) isolates had Bacteriophage Shelly (NC_041909.1), 1 out of 24 (4.17%) had *Bacillus* phage G (NC_023719.1) and 1 out of 24 (4.17%) isolates had incomplete *Streptococcus* phage 5093 (NC_012753.1) (Fig. 3).

### Pan-genome analysis and phylogenetic analysis of GBS genomes from elephants

**Fig. 4.**
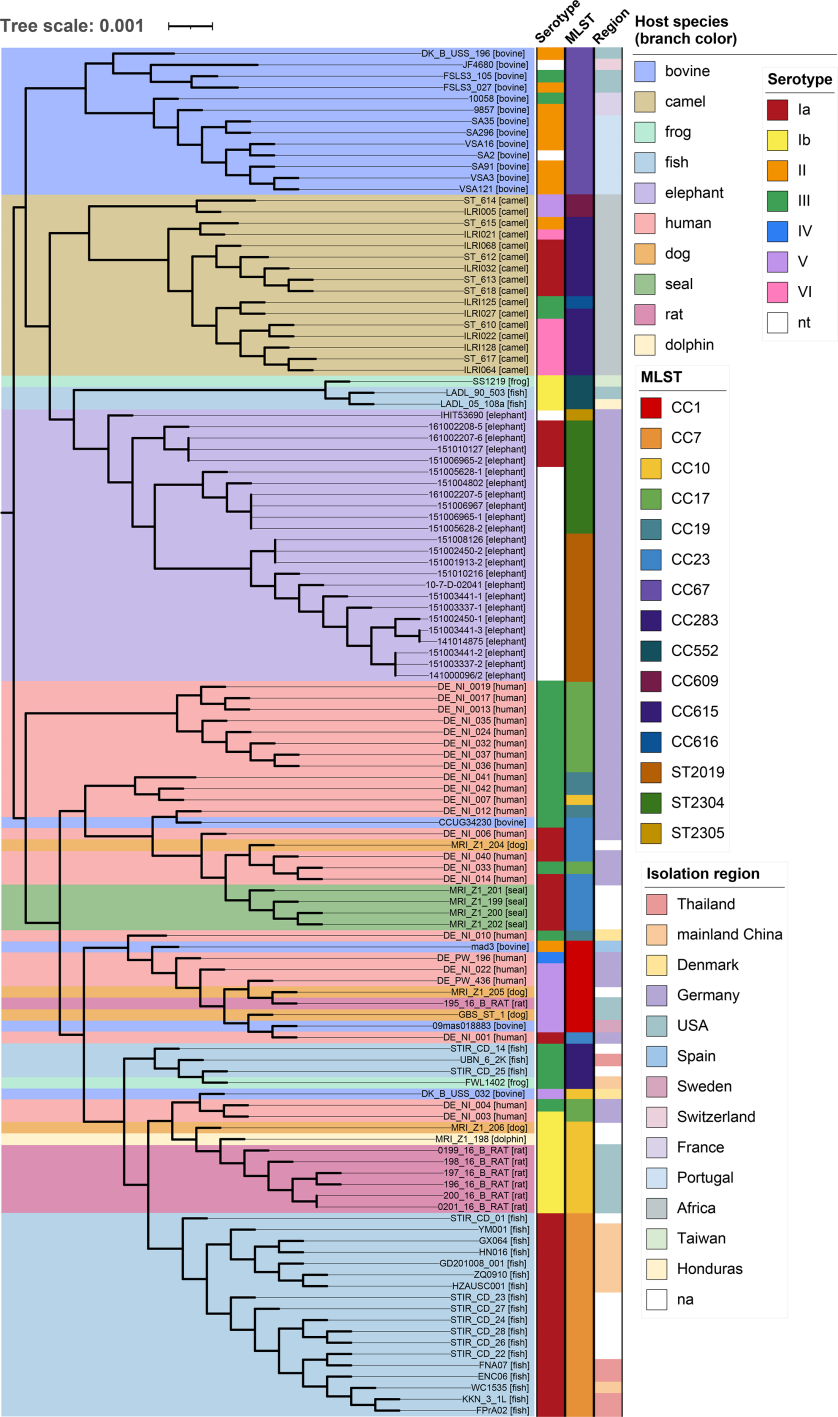
Phylogenetic analysis of different GBS isolates (*n*=121). The tree is colour-coded according to the respective host species, with the host species also indicated in square brackets. The scale bar corresponds to the number of amino acid substitutions per site, with branch lengths reflecting the relative genetic divergence among genomes. Annotation of the tree was done using iTOL.

**Fig. 5.**
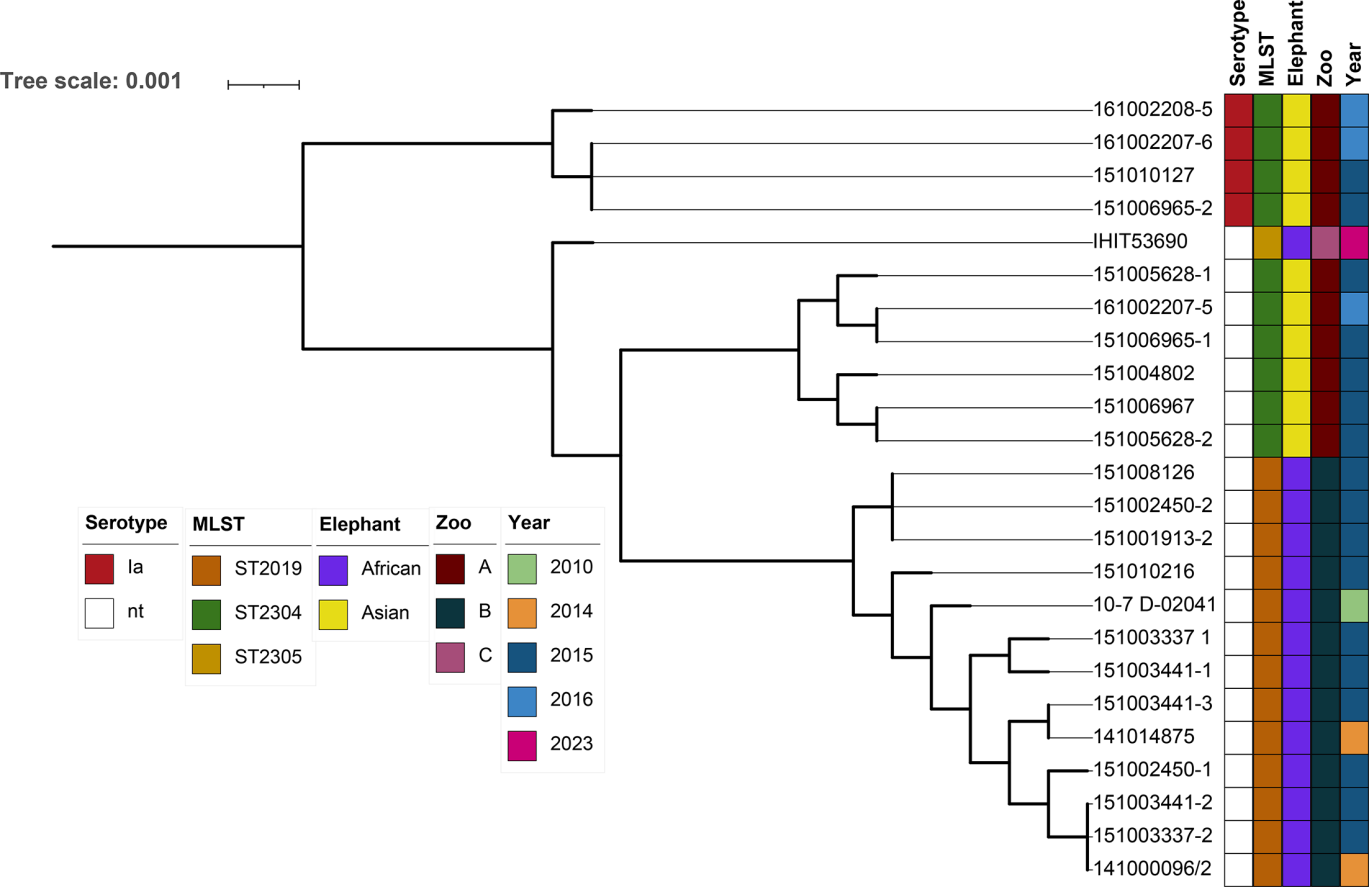
Core genome phylogenetic tree of the 24 elephant isolates. Coloured blocks showing capsular serotype, MLST, elephant species, zoo of origin and isolation year, as indicated by the legend. The scale bar corresponds to the number of amino acid substitutions per site, with branch lengths reflecting the relative genetic divergence among genomes. The tree was annotated using iTOL.

Analysis of the gene content of the 24 elephant-derived GBS isolates revealed a pan-genome comprising 2,352 genes, with a core genome of 1,629 genes. When the representative GBS genomes from other host species (as described in the ‘Method’ section) were included in the analysis, the pan-genome expanded to 5,485 genes, while the core genome decreased to 1,325 genes. Within the phylogenetic analysis, GBS isolates clustered into two broader clusters: one comprising lineages previously reported only in a single host species, such as camel-associated CC609, bovine-associated CC67 and the elephant sequence types identified in this study, and another cluster comprising lineages reported in multiple host species, including human-associated CC23, fish-associated CC7 and rat-associated CC10, and similar consistent with prior findings (Fig. 4) [52]. To further explore the genetic relationships among GBS isolates from elephants, a phylogenetic analysis was conducted exclusively on the 24 elephant-derived isolates (Fig. 5). The isolates generally clustered by geographical origin, corresponding to the zoo from which the host was sampled. An exception was the most recent isolate from an African elephant in Zoo C (collected in 2023), which shared a common ancestor with four GBS isolates from Asian elephants in Zoo A. Notably, a clear clustering pattern emerged based on both the zoo of origin and the collection year. In Zoo B, all isolates were from African elephants and shared the same sequence type (ST2019). These isolates lacked an identifiable capsular serotype and were primarily isolated in 2015. However, this clade also included two isolates from 2014 and one from 2010 that clustered very closely together, suggesting potential persistence of this GBS strain within the zoo population over multiple years.

With regard to Zoo A, isolates 161002208-5, 151010127, 151006965-2 and 161002207-6 formed a distinct clade separate from the other Zoo A isolates. Notably, these were the only isolates with an identifiable capsular serotype, all classified as serotype Ia. As expected, major clades aligned with MLST types: ST2019 isolates clustered together, while ST2304 clustered with the closely related ST2305 isolate, IHIT53690, as ST2305 is an SLV of ST2304 (Fig. 5).

### Comparison dataset cluster attribution and genome-wide association investigation

SKA identified a total of 96 clusters among all GBS genomes (*n*=121), with the elephant-derived isolates distributed across six distinct clusters (Table S3). However, the elephant isolates did not cluster with any isolates from other host species (Fig. S1). The median SKA SNP distance was 7,147.5 SNPs across all GBS isolates and 2,344.4 SNPs specifically for the elephant-derived isolates. Within Zoo A, the median SNP distance was 182.5 SNPs, while in Zoo B, it was 5.2 SNPs.

A genome-wide association study was conducted using a pan genome approach to investigate genes that are unique to GBS isolated from elephants. We have found that the *maR* gene was significantly associated with the elephant host, found in all elephant-derived isolates but not in any of the isolates from the other host species. Two additional proteins uniquely associated with all elephant isolates were the Maf family protein and the three acyl-CoA thioester hydrolase/BAAT C-terminal domain-containing proteins. The Lin0465 protein was found in all but one of the elephant isolates (IHIT53690) and was absent in all but two isolates from other host species (*P*-value: 8.17×10^−15^).

## Discussion

*S. agalactiae* is a known multi-host pathogen, primarily associated with infections in humans and bovines. However, it is far more frequently isolated as a harmless colonizer, with many strains representing asymptomatic carriage rather than disease. Despite this, GBS is also relatively frequently isolated in endangered species such as wild and zoo elephants. However, to date, no studies have investigated these GBS isolates from elephants using WGS.

The phylogenetic analysis demonstrates that the core genome of GBS isolates from elephants is distinct from GBS genomes isolated from different host species, supporting the niche specificity and potentially host-restricted lineage of GBS [52,53].

There were challenges in defining the elephant capsule type. In our study, serotype Ia was defined only in four GBS isolates, with the rest of the isolates classed as non-typable due to a deletion of multiple genes in the capsular locus. Recently, it has been reported that a human GBS isolate had deletions of multiple capsular genes [20], the same as identified in our study. Authors of the mentioned study have proposed that a recombination event led to the loss of the whole capsule locus, and large capsular recombination events are known to happen in the GBS population [54,55]. Given that elephants are a rarely reported host for GBS, as indicated by the limited studies available and, especially, only a small number of elephant GBS genomes available, further genetic and experimental data would be required to confirm if elephant GBS isolates are more likely to be acapsular as part of their species-specific adaptations. It is important to note that GBS isolates investigated here came from infections in elephants, and carriage isolates are not sought after; hence, we are not able to comment on the potential diversity of disease-causing vs asymptomatic or carriage strains of GBS in elephant populations. Notably, current development of GBS vaccines is primarily focused on human GBS vaccines based on ten capsular serotypes or specific surface proteins [56], and current vaccines for fish are also based on serotypes [57]. There is a concern that serotype-based vaccines, when implemented widely, will drive the evolution of non-vaccine serotype and acapsular GBS clones to emerge, similar to what has happened to pneumococcal clones [58]. The risk of this happening may increase if there is horizontal transfer of capsular operons between strains or even across different host species that occur more frequently [22,59].

Eleven of 24 GBS isolates from elephants had a novel ST2304 (*n*=10) and ST2305 (*n*=1), highlighting the genetic diversity in the GBS isolated from animals that is still unexplored. Notably, isolate IHIT53690 from Zoo C differed only by one allele variant from the isolates originating from Zoo B. The remaining GBS isolates from elephants were classified as ST2019 and were found only in Zoo B. Within the PubMLST database, only one other isolate of ST2019 has been reported, which was also reported as non-typable for its capsular serotype and has been isolated from elephants in the UK (personal communication, Dr Jauneikaite, manuscript in preparation). One of the limitations of our study is that there is potential confounding between host species and geographical location where the elephants were present at the time of sampling, as GBS isolates were available only from one species of elephants present in each of the zoo, potentially highlighting the genotype specificity linked to either elephant species or the zoo. Only one elephant species was present, and it is closely associated with the zoo of origin. Consequently, it is not possible to determine whether the observed STs are influenced primarily by host species or geographic and environmental factors. Furthermore, the dataset spanning over a time period of 13 years with irregular sampling intervals adds another layer of complexity to the interpretation of phylogenetic relationships and strain persistence. These limitations should be taken into account when interpreting findings related to host specificity, transmission patterns and the evolutionary trend of GBS in elephants and other species.

We have found the *mprF* gene present in all elephant-derived GBS genomes. This gene has been reported to play a role in bacterial resistance to cationic antimicrobial peptides, including agents like gentamicin or daptomycin, the latter classified as an antibiotic of last resort used in cases of multidrug-resistant infections [60]. It is known that GBS displays intrinsic resistance to gentamicin due to the low permeability of its cell wall to large molecules such as aminoglycosides [61]. The presence of the *mprF* gene may further contribute to reduced susceptibility, as it has been reported to affect the bacterial cell membrane and decrease susceptibility to certain antibiotic classes. In *Staphylococcus aureus*, for example, *mprF* is known to modulate susceptibility to cationic antibiotics, including the glycopeptide vancomycin, the aminoglycoside gentamicin and moenomycin [62,63].

PBPs, the enzymes essential for the synthesis of peptidoglycan as components of the cell wall in Gram-positive bacteria, have also been investigated in these isolates and found no mutations that had previously been reported to be linked with decreased susceptibility to penicillin or *β*-lactam antibiotics. Previous reports indicate that there are specific point mutations in PBPs, primarily PBP2x, that are linked to reduced susceptibility to *β*-lactam antibiotics [49,64]. We have found the A95D mutation in PBP1B and the V80A mutation in PBP2B in all elephant isolates, which has been previously reported, although they have only been tentatively linked to penicillin-non-susceptible GBS [49,50, 64]. None of the other substitutions in PBPs identified in this study have been associated with *β*-lactam resistance to date, and from limited phenotypic susceptibility data available to us, we were able to show that 12 GBS isolates investigated in our study did not show any evidence for resistance to penicillin [10].

Bacteriophages, including prophages, play a crucial role in the evolution of bacterial genomes and are often associated with enhanced infectivity, pathogenicity and virulence across various bacterial species [65,66]. Seven GBS isolates in our study carried intact prophage regions with most gene hit counts for *Streptococcus* phage T12. This phage is a prototypic temperate phage first associated with *Streptococcus pyogenes* that carries the *speA* gene coding for erythrogenic toxin A [67]. However, there are no reports of phage T12 in GBS, and none of the elephant isolates in the pan-genome analysis contained the *speA* gene or any related toxin.

When examining the presence of host-specific genes, only one gene was consistently found across all elephant GBS isolates in comparison to other GBS isolates. This gene encodes the MprF enzyme, responsible for the unique synthesis of a cationic glycolipid, Lys-Glc-DAG, which aids in the invasion of human endothelial cells, suggesting a potential role in enhancing bacterial entry into host cells and promoting disease progression [68]. However, it remains to be confirmed whether it fulfils the same function in elephants. The *maR* gene found exclusively in all elephant isolates encodes the MarR family transcriptional regulator, which plays a key role in controlling the oxidative stress response, a vital environmental sensing function, particularly for pathogenic bacteria [69]. A recent study postulated that the broad host range and ability of GBS to colonize different tissues may be due to the ability of its regulatory systems to recognize and respond to external stimuli, including oxidative and aerobic stress [70]. Maf-like proteins have been proposed to belong to a family of house-cleaning nucleotide hydrolyzing enzymes, which prevent the incorporation of noncanonical nucleotides into cellular DNA [71]. This could also offer an advantage in adapting to different hosts and environmental conditions. Lin0465, which encodes an intracellular PfpI protease in *Listeria*, plays a role in stress response [72,73]. A study postulated that lin0464, lin0465 and their homologues contribute more to the environmental fitness of these bacteria rather than to their virulence [73]. In summary, this set of genes unique to the elephant isolates examined here collectively confers advantages that enhance adaptability to various hosts and environmental conditions. By enabling GBS to respond effectively to diverse physiological and ecological challenges, these genes likely play a role in its ability to adapt to a host like the elephant and its survivability in different environments.

### Conclusion

GBS plays an important role in bacterial infections not only in human and bovine diseases, but also in less often studied host species like elephants. Comparative genomic analysis revealed that, in several aspects, the elephant GBS isolates differ from those of other host species, despite the geographical limitations of our study. Capsular typing of the elephant-derived isolates revealed a high proportion of non-typable strains due to a deletion in the *cps* locus, highlighting their genetic differentiation from serotypes predominantly characterized in human GBS isolates. To further clarify whether elephants may represent a distinct sublineage of GBS, future studies should include more isolates from elephants across diverse geographic regions. All of this could contribute to a better understanding of the zoonotic potential and pathogenic properties of GBS.

## Supplementary material

10.1099/mgen.0.001489 Fig. S1.

10.1099/mgen.0.001489 Table S1.

## Abbreviations

AMR: antimicrobial resistance
GBS: group B streptococcus
MLST: multilocus sequence typing
ONT: Oxford Nanopore Technologies
PBP: penicillin-binding protein
