# Supplementary material for: Evidence of a novel sublineage of Streptococcus agalactiae in elephants from zoo populations in Germany
Source: Microb Genom. 2025 Sep 4;11(9):001489. doi: 10.1099/mgen.0.001489 (PMC12421258; doi:10.1099/mgen.0.001489)
Supplement: Fig. S1. [file mgen-11-01489-s001.pdf]

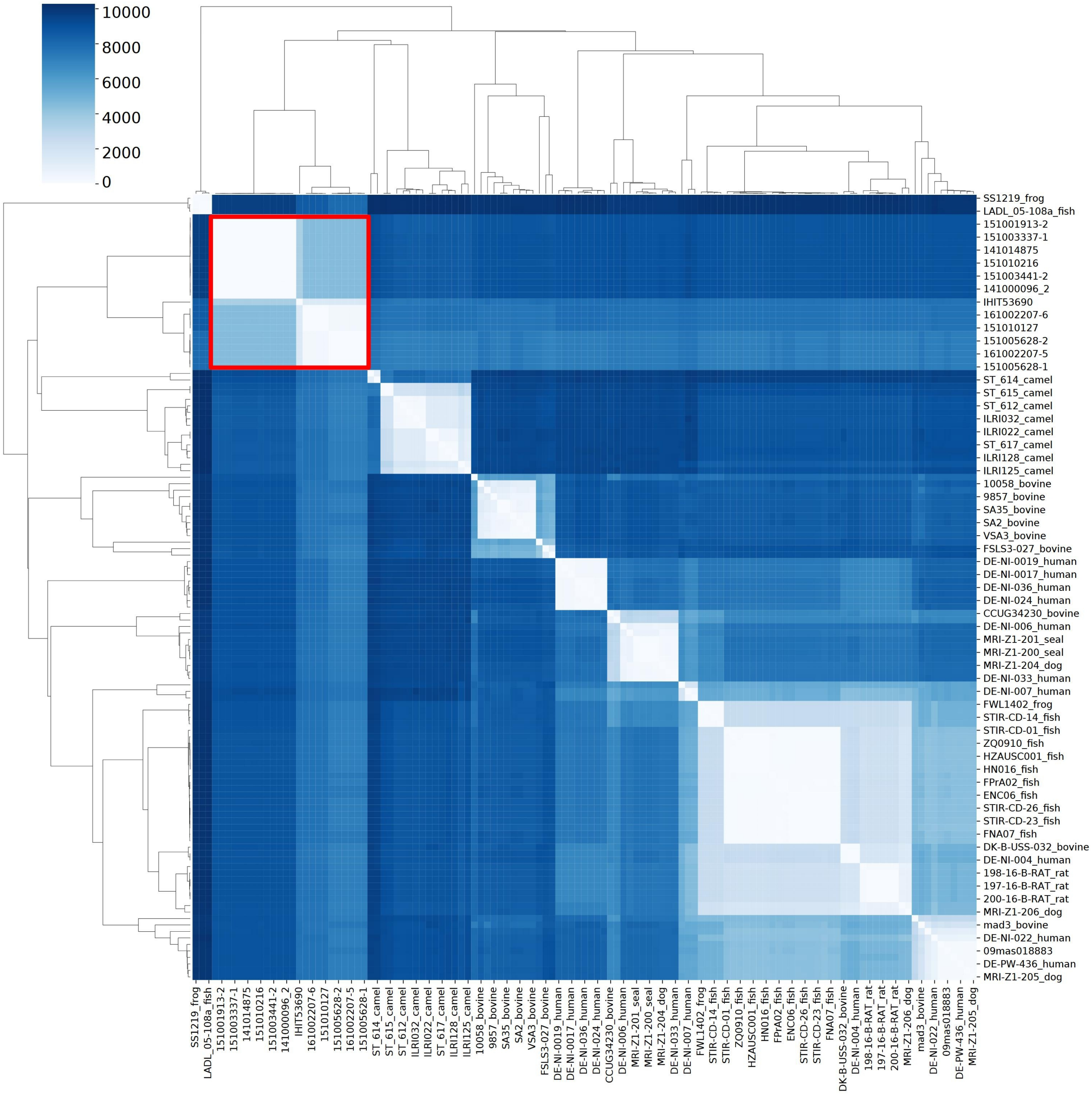

Supplementary Figure S1: Heatmap with dendrogram displaying the split *k*-mer clustering of various Group B streptococcus (GBS) isolates. The distance matrix was created using SKA with a 10 SNP threshold and a minimum identity cutoff of 0.9 and visualized with Python. The elephant cluster is marked with a red square.

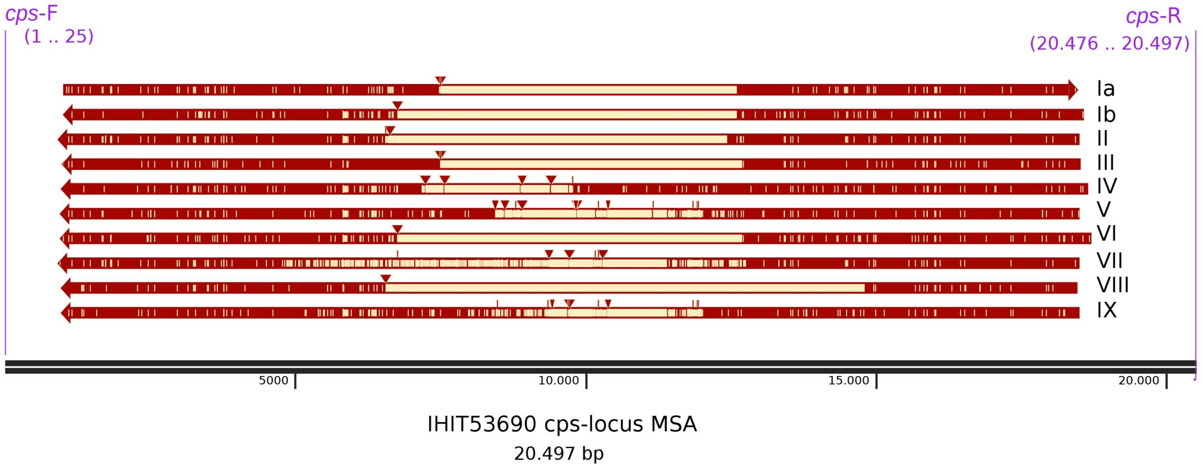

Supplementary Figure S2: Multiple-sequence alignment of IHIT53690 in comparison with the different capsular (*cps*) locus variants. The regions between the primers (*cps*-F and *cps*-R) are indicated in dark red, while the deletion is shown in beige. Extraction and alignment of the loci was done with SnapGene.
